# Uniform SiGe/Si quantum well nanorod and nanodot arrays fabricated using nanosphere lithography

**DOI:** 10.1186/1556-276X-8-349

**Published:** 2013-08-08

**Authors:** Hung-Tai Chang, Bo-Lun Wu, Shao-Liang Cheng, Tu Lee, Sheng-Wei Lee

**Affiliations:** 1 Institute of Materials Science and Engineering, National Central University, No. 300, Jhongda Rd., Jhongli 32001, Taoyuan, Taiwan; 2 Department of Chemical and Materials Engineering, National Central University, Jhongli 32001, Taiwan

**Keywords:** SiGe, Quantum wells, Quantum dots, Epitaxy, Nanosphere lithography, 61.72.uf, 62.23.Eg, 68.37.Lp, 68.65.Hb, 78.55.-m, 81.15.Kk

## Abstract

This study fabricates the optically active uniform SiGe/Si multiple quantum well (MQW) nanorod and nanodot arrays from the Si_0.4_Ge_0.6_/Si MQWs using nanosphere lithography (NSL) combined with the reactive ion etching (RIE) process. Compared to the as-grown sample, we observe an obvious blueshift in photoluminescence (PL) spectra for the SiGe/Si MQW nanorod and nanodot arrays, which can be attributed to the transition of PL emission from the upper multiple quantum dot-like SiGe layers to the lower MQWs. A possible mechanism associated with carrier localization is also proposed for the PL enhancement. In addition, the SiGe/Si MQW nanorod arrays are shown to exhibit excellent antireflective characteristics over a wide wavelength range. These results indicate that SiGe/Si MQW nanorod arrays fabricated using NSL combined with RIE would be potentially useful as an optoelectronic material operating in the telecommunication range.

## Background

Over the past decades, there has been enormous interest in fabricating periodic semiconductor nanostructures, in which the semiconductor nanodot or nanorod array has shown its great potential for future applications in photonic crystals [1], nanoscale transistors [2], field electron emitters [3], biomaterials [4], and light-emitting devices [5]. The well-known top-down techniques providing accurate size and geometric control in periodic semiconductor nanostructure patterning include laser interference lithography [6], nanoimprint lithography [7], ion beam lithography [8], and electron beam lithography [9]. However, the cost and complexity of these techniques increase dramatically with the demand for reduced feature sizes over large areas. Nanosphere lithography (NSL) has emerged as an alternative nanofabrication technique, where a monodisperse or multidisperse nanosphere template acts as an etching or deposition mask to transfer its pattern to the underlying substrate [10-12]. The sizes of nanospheres can be tuned from 20 to 1,000 nm [13,14], offering a simple and inexpensive solution to scale nanostructure feature sizes. More importantly, the location, density, and coverage of nanostructures can be well controlled. With improvements in the domain sizes of the self-assembled nanosphere arrays [15], NSL has great potential in fabricating nanoscale electronics, optoelectronics, thermoelectrics, and biosensors. Over the past decade, NSL has been used to nanopattern Si [16], GaAs [17], and glass [18] substrates. Recently, we also demonstrated the realization of SiGe nanorod arrays on SiGe virtual substrates using NSL combined with catalytic etching [19].

On the other hand, the idea of integrating optoelectronic and electronic devices into Si chips has always been highly attractive due to the benefits in cost, reliability, and functionality [20]. However, Si is an indirect bandgap semiconductor and thus of limited use for optoelectronic applications. Many efforts have been made to resolve the low quantum efficiency of Si associated with its indirect bandgap. One important approach is the combination of Si with other semiconductor materials, such as Ge or Si_1 − *x*_Ge_*x*_ alloys for heterostructures. For this purpose, Si/Ge superlattices (SLs) [21], multiple quantum wells (MQWs) [22], and multiple quantum dots (MQDs) [23] have been demonstrated to adjust the bandgap and reduce nonradiative recombination. Choi et al. further reported that the formation of microdisks from the Si/Ge/Si single QW using electron beam lithography significantly enhanced the intrinsic photoluminescence (PL) transitions [9]. Chen also fabricated pyramidal nanodots that possess Si/Ge SLs by chemical selective etching through a self-assembled Ge QD nanomask and found an obvious enhancement in PL emission [24]. In addition, an improvement of light extraction from SiGe/Si MQWs with nanowall structures fabricated by electron cyclotron resonance plasma etching through a random Al-masked pattern was also reported [25]. However, few studies reported the fabrication of periodic nanostructure arrays composed of SiGe/Si MQWs using NSL. In this study, we demonstrate the fabrication of optically active uniform SiGe/Si MQW nanorod and nanodot arrays from the Si_0.4_Ge_0.6_/Si MQWs using NSL combined with the reactive ion etching (RIE) process. Compared to the as-grown sample, we observe an obvious blueshift in PL spectra for the SiGe/Si MQW nanorod and nanodot arrays, which can be attributed to the transition of PL emission from the upper multiple quantum dot-like (MQD-like) SiGe layers to the lower MQWs. In addition, the SiGe/Si MQW nanorod arrays are also shown to exhibit excellent antireflective characteristics over a wide wavelength range.

## Methods

**Figure 1:**
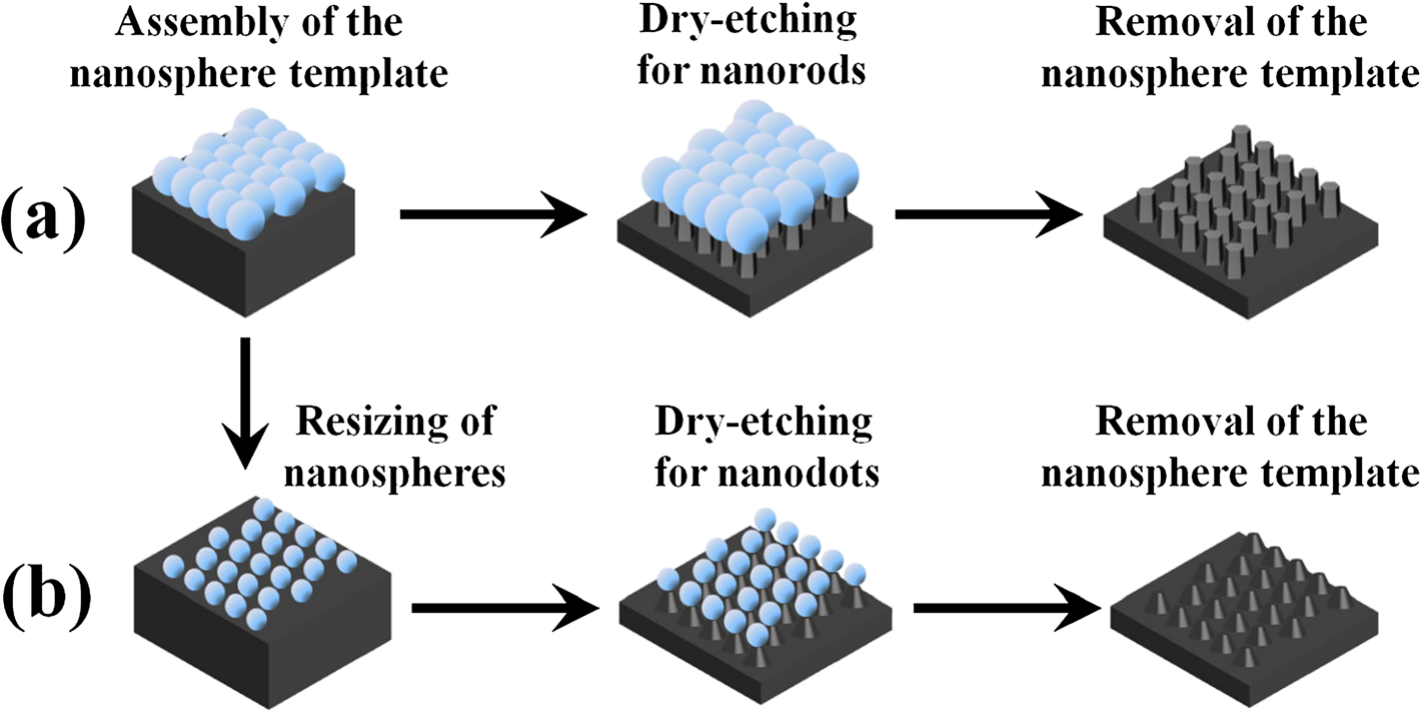
**Schematic of the experimental procedure.** To fabricate uniform SiGe/Si MQW **(a)** nanorod and **(b)** nanodot arrays from the Si_0.4_Ge_0.6_/Si MQWs using NSL combined with the RIE process.

Our initial samples consist of 50-period Si_0.4_Ge_0.6_/Si (3.6/6.4 nm nominally) MQWs capped with a 50-nm-thick Si layer, which were grown on (001) Si wafers using a multi-wafer ultra-high vacuum chemical vapor deposition (UHV/CVD) system. Pure SiH_4_ and GeH_4_ were used as gas precursors for Si or SiGe epitaxy. The formation procedure of SiGe/Si MQW nanorod arrays from the SiGe/Si MQW samples is illustrated in Figure 1(a): (1) assembly of the polystyrene (PS) nanosphere monolayer arrays, (2) etching of the SiGe/Si MQW samples by RIE, and (3) removal of the nanosphere template. For the formation of SiGe/Si MQW nanodot arrays, PS nanosphere arrays were first resized and then used as an etching mask, as shown in Figure 1(b). The following is a detailed introduction of the fabrication procedure.

It is crucial to obtain a hydrophilic surface to allow the self-assembly of PS nanosphere monolayer arrays. In the first step, the as-grown SiGe/Si MQW samples were ultrasonically cleaned in acetone and in a solution of 4:1 H_2_SO_4_/H_2_O_2_ at 80°C for 30 min to prepare a hydrophilic surface. The SiGe/Si MQW samples were then coated with 800-nm-diameter PS nanospheres to form highly ordered and close-packed nanosphere arrays. Subsequently, a mixture of SF_6_ and O_2_ was used to etch the samples at a working pressure of 25 mTorr for various durations to form the SiGe/Si MQW nanorod arrays. During the RIE etching, the inductively coupled plasma (ICP) power and bias of the etcher were kept at 50 W and 25 V, respectively. Finally, the PS nanosphere template was removed by ultrasonically cleaning in acetone solution. In addition, for the nanosphere resizing, O_2_ plasma RIE was used to shrink the PS nanospheres, allowing postspin feature size control.

The surface morphologies of the etched samples were examined by scanning electron microscopy (SEM; FEI Quanta 200F, Hillsboro, OR, USA). Transmission electron microscopy (TEM) was carried out with a JEOL 2100 TEM (Akishima, Tokyo, Japan) operating at 200 kV to reveal detailed information about the microstructures of the etched nanostructures. PL measurements were performed at 10 K to study the optical properties of the SiGe/Si MQW nanorod and nanodot arrays using a 514.5-nm line of an Ar^+^ laser. The PL spectra were recorded by a liquid nitrogen-cooled Ge photodetector with the standard lock-in technique. We also measured total hemispherical reflectance spectra in air on a spectrophotometer with an integrating sphere (300 to 2,000 nm, Hitachi U-4100, Chiyoda, Tokyo, Japan) for the etched SiGe/Si MQW nanostructures.

## Results and discussion

**Figure 2:**
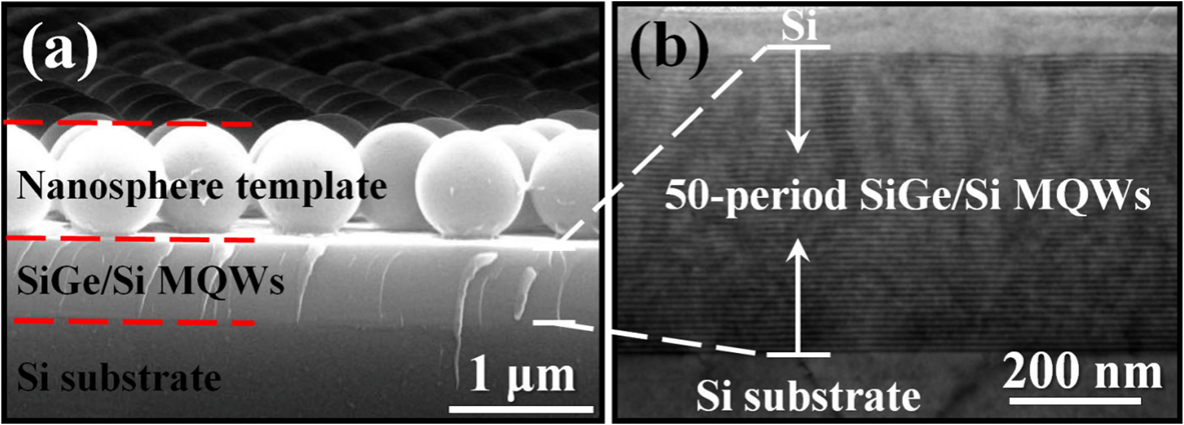
**SEM and TEM images of the starting SiGe/Si MQW sample. (a)** SEM image showing an 800-nm-diameter PS nanosphere monolayer coated on the SiGe/Si MQW sample. **(b)** Cross-sectional TEM image showing the 50-period SiGe/Si MQWs epitaxially grown on Si.

**Figure 3:**
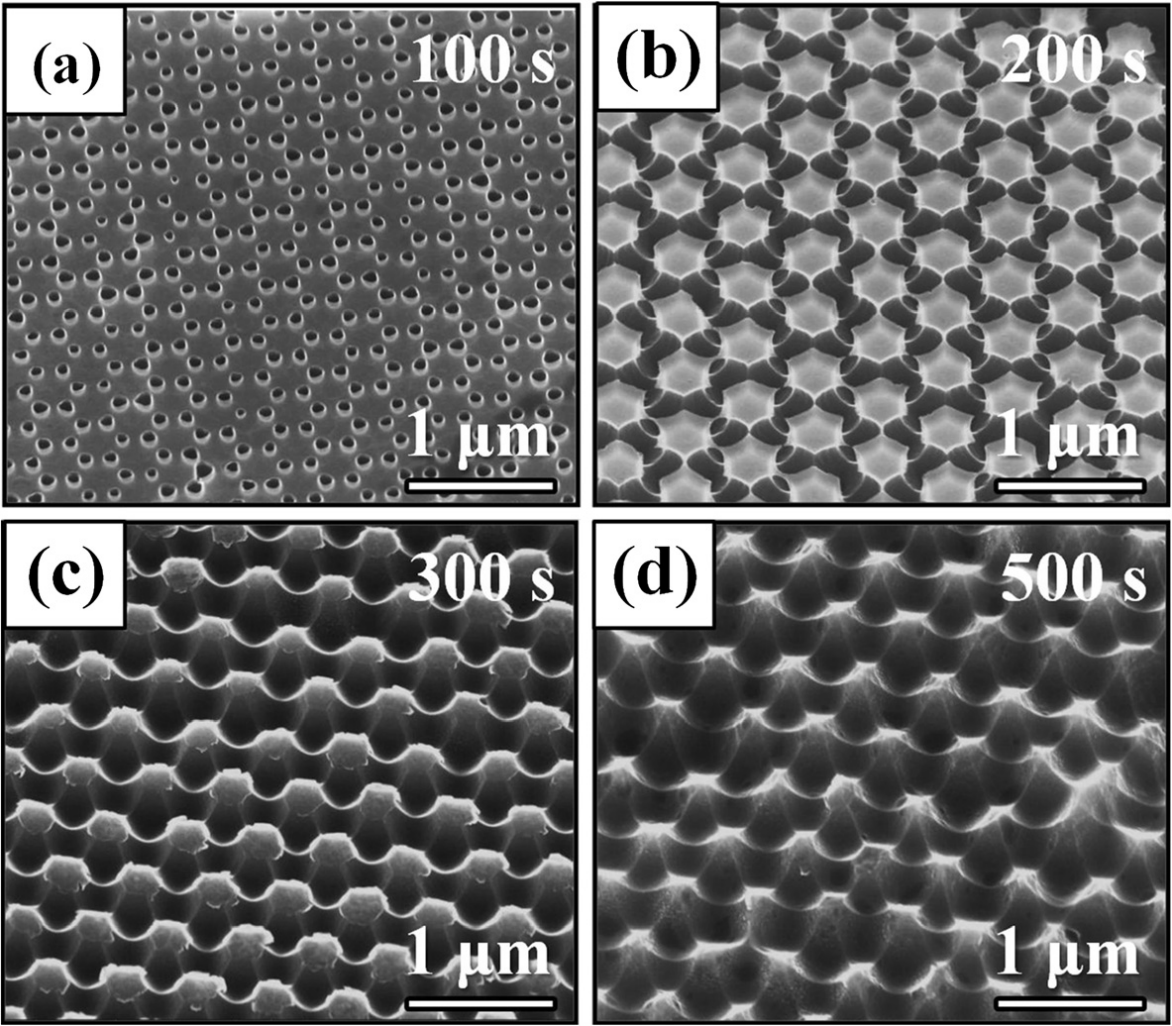
**SEM images of the SiGe/Si MQW samples etched by RIE for different durations. (a)** 100 s, **(b)** 200 s, **(c)** 300 s and **(d)** 500 s, respectively.

Figure 2a shows a typical SEM image of a monolayer colloidal crystal consisting of 800-nm-diameter PS nanospheres on the SiGe/Si MQW sample. It is evident that the coated PS nanospheres are hexagonal close-packed ordering. Figure 2b also shows the cross-sectional TEM image for the SiGe/Si MQWs. No defects such as threading dislocations were observed within the SiGe/Si MQWs even if extending the observation area, indicating the high-quality SiGe epitaxy by UHV/CVD. In the following RIE process, the etching rate of Si or SiGe with a mixture of SF_6_ and O_2_ is much higher than that of PS nanospheres. Therefore, the nanosphere template acts as an etching mask, and a variety of SiGe/Si MQW nanostructures can be produced using RIE. At the beginning of the etching process shown in Figure 3a, the nanopits were formed at the vertex of a hexagon on the surface, indicating that the PS nanospheres indeed acted as an etching mask and the unprotected surface (i.e., the interstices of nanospheres) was preferentially etched by the reactive F ions during the RIE process. With increasing etching times to 200 and 300 s (Figure 3b, c), the pattern of the nanosphere template was successfully transferred to the underlying substrate to form the close-packed nanorod arrays. The preservation of the hexagonal ordering and the interdistance of the original nanospheres are apparent for the resulting nanorod arrays. With further increase in etching time to 500 s, these nanorod arrays finally transformed into the pyramid-like nanostructures (nanopyramids) with the reduced heights (see Figure 3d).

**Figure 4:**
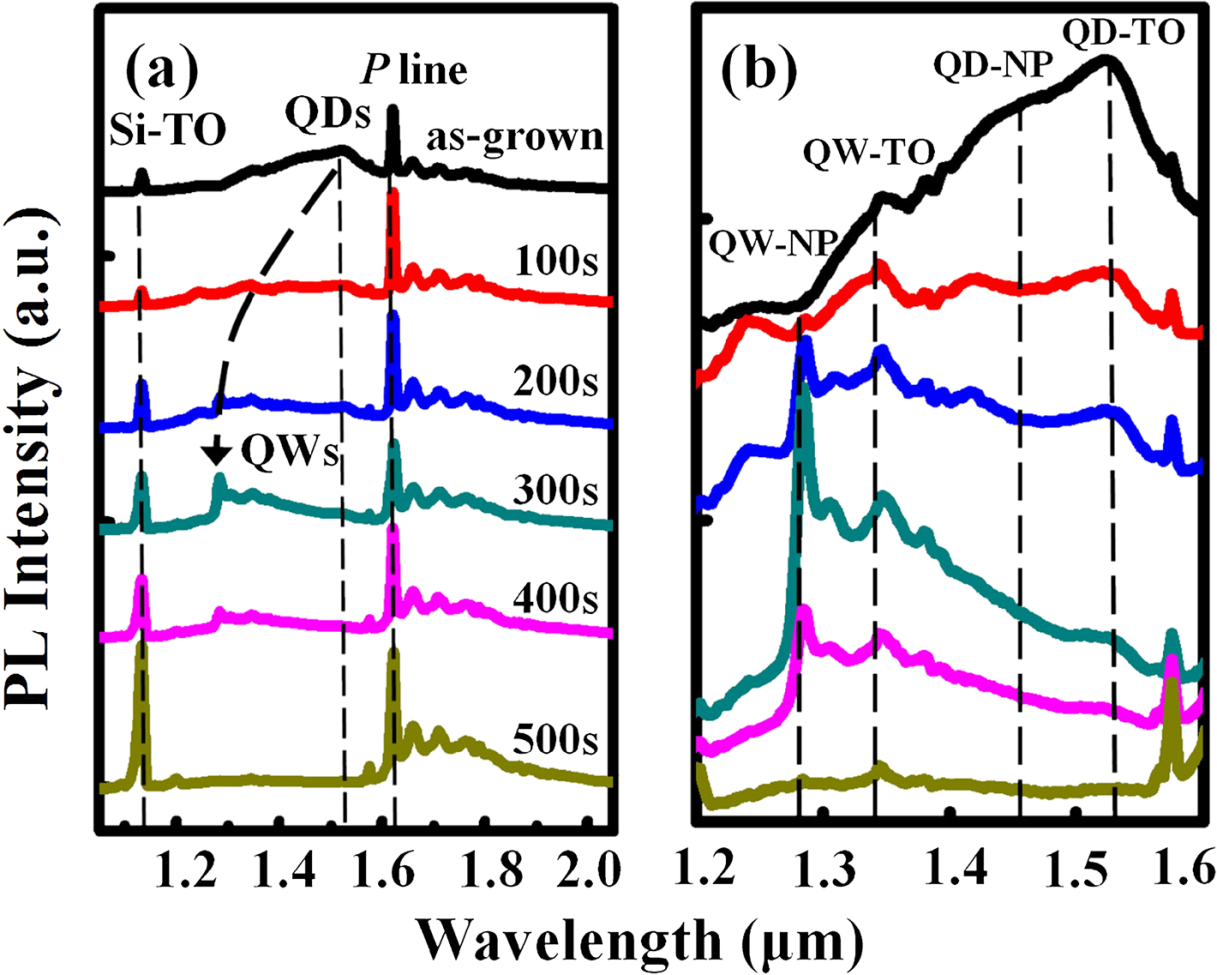
**PL spectra measured at 10 K of the as-grown and etched samples. (a)** PL spectra in the wavelength range from 1.0 to 2.0 μm of the as-grown and etched SiGe/Si MQW samples with different etching times. **(b)** PL spectra in the wavelength range from 1.2 to 1.6 μm are amplified.

Figure 4a shows the corresponding PL spectra measured at 10 K. The narrow peak located at 1.62 μm (namely, the *P* line) with its satellites at longer wavelengths arises from the C-O complexes in Si as reported for many different SiGe structures [26,27]. The strong peak around 1.1 μm is assigned to the transverse optical (TO) phonon-assisted recombination in bulk Si. Therefore, the peaks between the Si TO peak and *P* line, which are amplified as shown in Figure 4b, can be attributed to the PL emissions from the SiGe/Si MQWs. First, we observe that the as-grown SiGe/Si MQW sample exhibits a very broad PL emission in the range from 1.3 to 1.55 μm, similar to the near-bandgap transition in Ge/Si MQDs [28]. This broad peak could be further deconvoluted into two main Gaussian line-shaped peaks at 1.45 and 1.52 μm, respectively. The higher-energy peak can be assigned to the no-phonon (NP) transition resulting from recombination of the bound exciton without phonon participation, and the lower-energy peak is the TO replica of Si_1 − *x*_Ge_*x*_ alloys [28,29]. In addition, some weak peaks located around 1.4 μm can be assigned to the dislocation-related PL lines, the so-called *D* lines, which have been widely observed in SiGe heterostructures [30]. With an appropriate etching time (300 s here) to form the nanorod arrays, the main PL peak is blue-shifted to the position of 1.28 μm and then gradually diminishes with further increasing etching time. This peak position is very close to that of the *G* line due to carbon contamination in bulk Si [31]. However, we can exclude this possibility since the intensity of this peak shows no obvious trend with the etching times. We also exclude the possibility of quantum confinement-related PL blueshift because the mean dimension within the growth plane of the nanorods (approximately 500 nm) apparently exceeds the critical size (usually below 10 nm) for the quantum confinement effect. Thus, two peaks located at 1.28 and 1.35 μm are believed to correspond to a NP transition and an associated TO phonon replica from the SiGe/Si MQW nanorod arrays.

**Figure 5:**
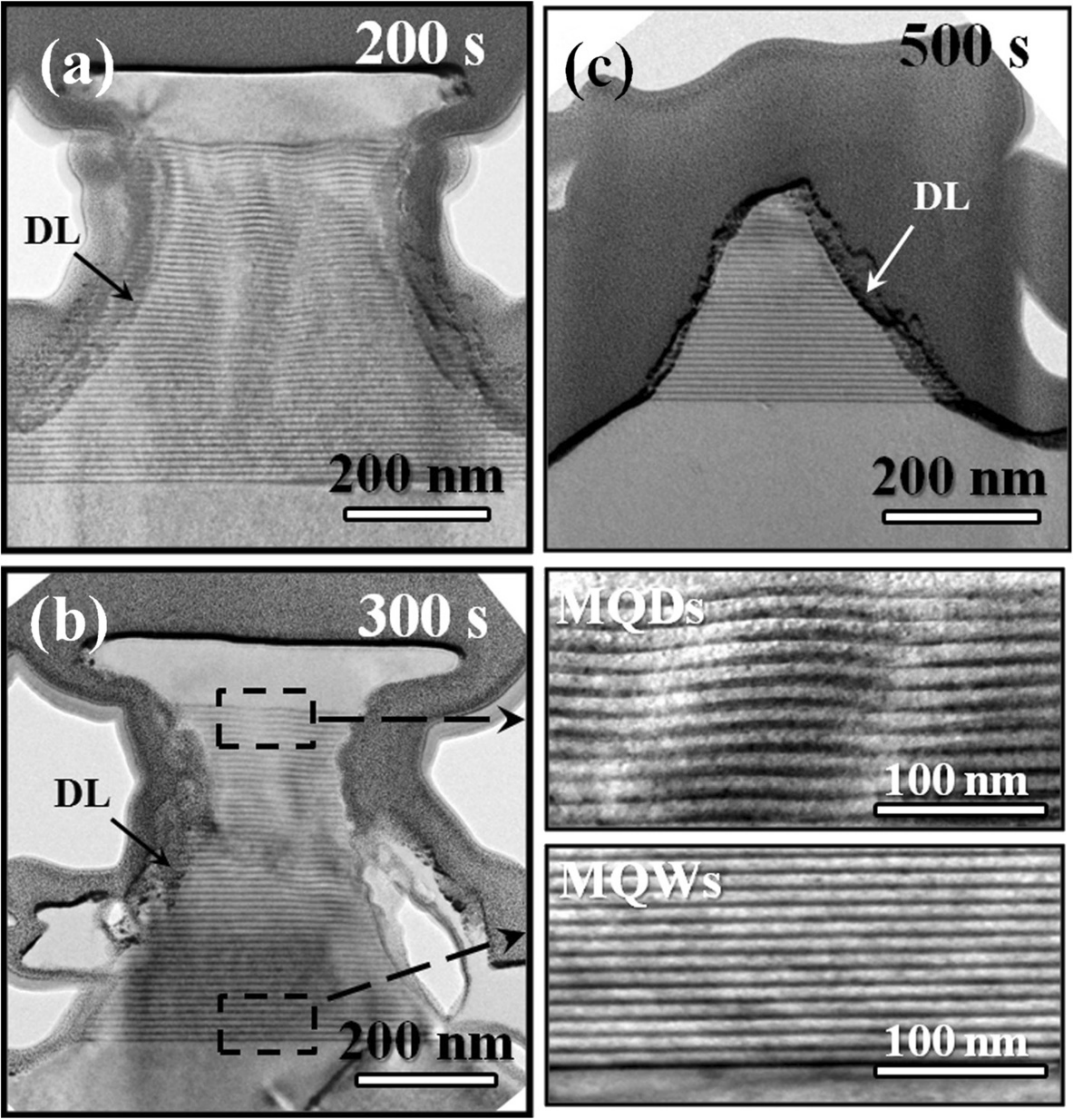
**Cross-sectional TEM images for the etched SiGe/Si MQW samples.** The samples were etched for **(a)** 200 s, **(b)** 300 s and **(c)** 500 s, respectively. The right column of (b) also provides the high-magnification view for the upper and lower SiGe layers within a SiGe/Si MQW nanorod, respectively.

We attempt to interpret this PL transition with the TEM observations. The TEM image shown in Figure 5a indicates that the sample etched for 200 s exhibits the sandglass-like nanorods, which consist of the complete 50-period SiGe/Si MQWs. With further increase in etching time to 300 s, the nanorods still retain the sandglass-like structure, but their lateral diameter becomes much smaller (see Figure 5b). The right column of Figure 5b further shows the high-magnification TEM images for the upper and lower SiGe layers within the SiGe/Si MQW nanorods, respectively, revealing two different layer features. While the lower SiGe layers retain an explicit QW structure, the upper SiGe layers reveal a MQD-like feature. It is well known that epitaxial growth of Ge or SiGe with high Ge content onto Si leads to a strain-induced spontaneous formation of the three-dimensional QDs as the epilayer exceeds a critical thickness, which is the so-called Stranski-Krastanov growth mode [32,33]. We can imagine that the upper SiGe layers in the MQWs are highly strained during the epitaxial growth and thus tend to form SiGe QDs to relieve the accumulated strain. Many studies have proposed the type II band alignment for both SiGe/Si MQW and MQD structures [34,35]. In a type II alignment, the indirect excitons are first localized at the hetero-interfaces and then recombine. Generally, the SiGe QDs are thought to be locally SiGe-alloyed and exhibit a dot size distribution [36,37]. Hence, a broad PL emission contributed from the upper SiGe layers of the as-grown sample can be expected, as shown in Figure 4b. On the other hand, although the lower SiGe layers in the MQWs retain a well-defined QW structure, they suffer from a higher degree of SiGe intermixing than the upper ones due to the longer annealing period in growth, thus resulting in the reduction of valence band offset in SiGe/Si structures. When the SiGe/Si MQW nanorods are formed by RIE, the lower SiGe layers are optically activated due to the favorable geometry of nanorods. A strong and sharp PL emission with an obvious blueshift is observed in the PL spectra for the SiGe/Si MQW nanorods. However, with further increase in etching time to form the MQW nanopyramids (Figure 5c), this PL peak diminishes due to the severe material loss after the RIE process.

In Figure 4b, we also find that in spite of the large material loss in the RIE process, the SiGe/Si MQW nanorod arrays exhibit a strong PL intensity comparable to that of the as-grown counterpart. We suggest that there exists a possible mechanism for PL enhancement. As mentioned above, this PL enhancement is difficult to be attributed to quantum confinement or indirect–direct bandgap transition since the mean diameter of the MQW nanorods is much larger than the exciton Bohr radius of Si and Ge. Some groups have reported the enhancement of PL intensity by laterally patterning the III-V or IV-IV heterostructures with the sizes similar to or larger than that in this study. A significant enhancement of the quantum efficiency in the PL spectra has been observed by forming GaN/AlGaN MQW microdisks of about 9-μm diameter and interpreted as a suppression of impurity-related transitions [38]. Choi et al. also associated the PL enhancement with carrier localization in the 500- and 1,000-nm-diameter Si/Ge/Si microdisks fabricated by electron beam lithography, the existence of which suppresses impurity-related nonradiative combination [9]. The similar mechanism may also contribute to the enhancement of PL intensity in our SiGe/Si MQW nanorod arrays. In addition, in this study, the high-density plasma generated during RIE process may severely damage the surface of SiGe/Si MQW nanorods and therefore form a 10- to 20-nm-thick amorphized layer on the surface. This may result in the formation of an effective ‘dead layer’ (indicated by DL in Figure 5a, b, c), in which nonradiative recombination processes dominate. This dead layer will further reduce the effective lateral size of the nanorods because carriers able to participate in optical process are confined to the undamaged region of the MQW nanorods. This factor may also act in the PL emission process and further enhance the PL intensity. For practice applications, this dead layer can be minimized by adjusting the RIE process parameters, such as reducing ICP power, decreasing reactive gas fluxes, and lowering the operating temperature. In addition, this damaged layer can be removed by an etchant [39].

**Figure 6:**
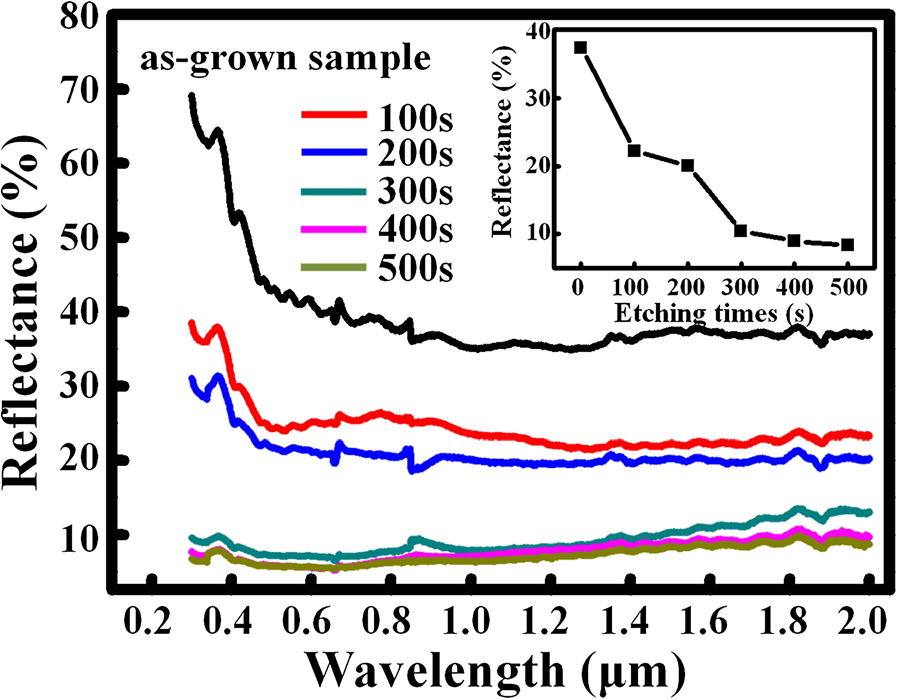
**Optical reflection spectra with wavelengths from 0.3 to 2 μm for the as-grown and etched samples.** The spectra were measured at an incident angle of 5°. The inset also shows the variation in reflectance at 1.55 μm as a function of etching times.

We also observe that the coverage of the etched samples decreases upon increasing the RIE durations (from nanopits, nanorods, and finally to nanopyramids), leading to the different roughness values. Optical reflectance has been a sensitive nondestructive method to examine the etched surface morphology. Figure 6 shows the optical reflection spectra with wavelengths from 0.3 to 2 μm for the as-grown and etched samples. The inset in Figure 6 is also a plot showing the variation of reflectance at 1.55 μm as a function of etching times. The reflectance is found to monotonically decrease with the etching times. The SiGe/Si MQW nanorod sample (i.e., the sample etched for 300 s) show considerably low reflectance over a wide wavelength, only 7.1% and 10.5% at 0.6 and 1.55 μm, respectively. This excellent antireflective characteristic can be attributed to its highly roughened surface. Many techniques including laser- [40] and metal-assisted [41] chemical etching have been reported to fabricate ‘black silicon’ with an ultra-low reflectance. The surface nanoroughening process in this study could be an alternative approach applied to SiGe-based nanodevices and optoelectronics, such as metal-oxide-Si tunneling diodes [42], light-emitting diodes [25], and photodetectors operating in the telecommunication range [28]. In addition, the SiGe/Si MQW nanopits and nanorods with well-defined spatial periodicity fabricated in this study would also be potential materials applied to photonic crystals [1] and phototransistors [43].

**Figure 7:**
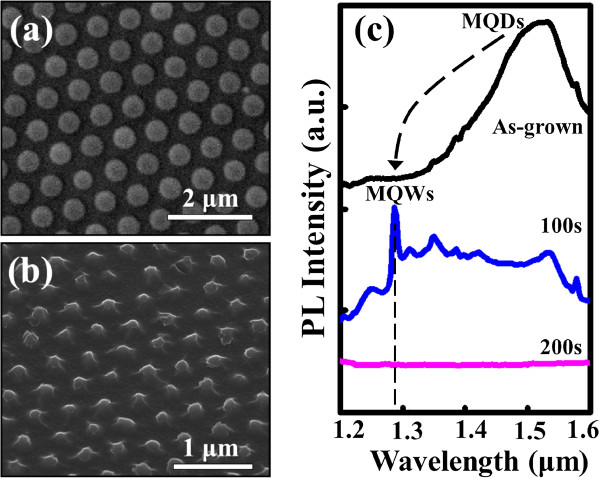
**SEM images and PL spectra of the etched MQW samples using a resized nanosphere template.** SEM images showing **(a)** the resized nanospheres with a mean diameter of approximately 480 nm and **(b)** the resulting SiGe/Si MQW nanodot arrays. **(c)** PL spectra in the wavelength range from 1.2 to 1.6 μm of the as-grown and etched SiGe/Si MQW samples fabricated using a resized nanosphere template.

Following the slimier fabrication processes, we can also produce the SiGe/Si MQW nanodots through a resized nanosphere template (Figure 7a). With an appropriate etching time (100 s here), the nanodot arrays consisting of several-period SiGe/Si MQWs can be obtained (Figure 7b). As shown in Figure 7c, although the characteristic PL emission from the MQW nanodot arrays also shows a similar blueshift relative to the as-grown sample, its peak intensity is apparently weaker than that of the as-grown sample possibly due to the severe material loss in the RIE process. We believe that by properly adjusting the process parameters of RIE, the PL characteristics of the MQW nanodots can be improved. Nevertheless, all of these nanofeatures contribute to the potential applications of using NSL combined with RIE to laterally nanopattern SiGe/Si heterostructures.

## Conclusions

In conclusion, this study demonstrates the fabrication of optically active uniform SiGe/Si MQW nanorod and nanodot arrays from the Si_0.4_Ge_0.6_/Si MQWs using NSL combined with reactive RIE. Compared to the as-grown sample, we observe an apparent blueshift in PL spectra for the SiGe/Si MQW nanorod and nanodot arrays, which can be attributed to the transition of PL emission from the upper MQD-like SiGe layers to the lower MQWs. A possible mechanism associated with carrier localization is proposed for the PL enhancement. Moreover, the SiGe/Si MQW nanorod arrays are shown to exhibit excellent antireflective characteristics over a wide wavelength range from the ultraviolet to infrared. This work offers a low cost and feasible alternative for designing and fabricating SiGe/Si nanostructured arrays as a potential material of multifunctionality.

## Competing interests

The authors declare that they have no competing interests.

## Authors’ contributions

H-TC prepared all SiGe/Si MQW samples and conducted the material characterizations. B-LW performed the NSL and RIE experiments. S-LC conducted the reflectance measurements. TL provided the polystyrene nanospheres. S-WL designed the study, analyze the data, and wrote the manuscript. All authors read and approved the final manuscript.

## Authors’ information

H-TC is currently a Ph.D. candidate of National Central University (Taiwan). B-LW is a Master's degree student of National Central University (Taiwan). S-LC and TL are professors of the Department of Chemical and Materials Engineering at National Central University (Taiwan). S-WL is an associate professor of the Institute of Materials Science and Engineering at National Central University (Taiwan).
